# Pre-packaged food targeted to gastrointestinal pathologies: are they low in FODMAP?

**DOI:** 10.1017/S000711452510528X

**Published:** 2025-11-14

**Authors:** Sara Barreirinhas, Carla Almeida, Susana Casal, Patrícia Padrão

**Affiliations:** 1 Faculdade de Ciências da Nutrição e Alimentação da Universidade do Porto, Porto, Portugal; 2 Faculdade de Ciências da Universidade do Porto, Porto, Portugal; 3 EPIUnit – Instituto de Saúde Pública da Universidade do Porto, Porto, Portugal; 4 Laboratório para a Investigação Integrativa e Translacional em Saúde Populacional (ITR), Porto, Portugal; 5 LAQV-REQUIMTE, Laboratório de Bromatologia e Hidrologia, Faculdade de Farmácia, Universidade do Porto, Porto, Portugal

**Keywords:** Carbohydrates, FODMAP diet, Food composition, Irritable bowel syndrome, Functional food

## Abstract

A diet low in fermentable carbohydrates, oligosaccharides, disaccharides, monosaccharides and polyols (FODMAP) has been described as an effective nutritional approach in irritable bowel syndrome. There has been an increased demand for gluten- and lactose-free foodstuffs in the last few years, which are associated with gastrointestinal symptoms and are consumed by patients with gastrointestinal disease. This study aims to estimate the FODMAP content of industrial pre-packaged food products containing the ‘gluten-free’ and ‘lactose-free’ claims. The ingredient lists of the foodstuffs from a Portuguese food retail chain were verified and classified according to their estimated FODMAP content as ‘low’ and ‘high’, using as a reference the FODMAP cutoff values and the serving sizes established by the Monash University App. Descriptive statistics and the Pearson *χ* ^2^ test were applied. From the 436 eligible products identified, most were classified as ‘low’ (53·0 %, *n* 231), 24·3 % (*n* 106) were classified as ‘high’ and 22·7 % (*n* 99) were classified as having ‘unknown’ FODMAP content. ‘High FODMAP’ products accounted for 12·2 % of those with ‘lactose-free’ claims and 31·6 % of those with ‘gluten-free’ claims. The ‘ready meals’ and ‘sauces, dressings, creams and soups’ were the food categories with the highest proportion of products with high FODMAP sources. This study showed that approximately a quarter of pre-packaged industrial foods targeted to gastrointestinal pathologies are high in FODMAP. The nutritional information on the label should be more specific, enabling more accurate dosing of FODMAP contents in foodstuffs and the establishment of the recommended serving sizes.

## Graphical abstract

**Figure:**
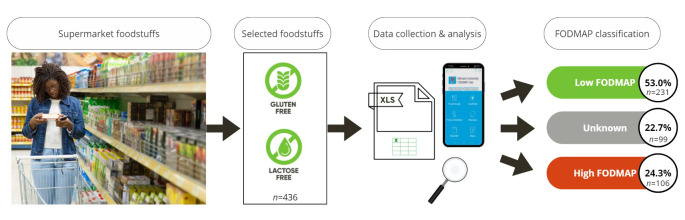

Irritable bowel syndrome (IBS) is recognised as a gut–brain interaction disorder, and its incidence has been increasing in recent years^(1,2)^. IBS is characterised by a set of gastrointestinal (GI) and extra-intestinal signs and symptoms that are often debilitating and negatively affect the quality of life^(3,4)^.

A diet low in fermentable carbohydrates, oligosaccharides, disaccharides, monosaccharides and polyols (FODMAP) has been described as an effective nutritional approach to reduce GI symptoms, as they appear to aggravate the symptomatology of various digestive conditions, including the IBS^(3,5)^. FODMAP are naturally present in several foods, including fruits, vegetables, cereals and dairy products, and are also used as processing ingredients by the food industry, added to improve the sensorial or technical quality, such as fructose, lactose or polyols^(6,7)^.

The low FODMAP diet prescription and monitoring by a qualified dietitian is essential for the patient’s therapy during three distinct phases after diagnosis^(2,8–10)^. In the first phase, high FODMAP foods are restricted, favouring only low FODMAP foods as alternatives^(2,3,8,10)^. High FODMAP foods should be gradually reintroduced in the second phase^(2,3,9,10)^. In the third and last phase, the diet should be personalised, according to the triggers of symptoms identified and the foods well tolerated by the individual^(2,8,9)^. The choice of commercial foodstuffs remains challenging, however, given the lack of detail on the label regarding FODMAP composition. In addition, both dietitians and patients deal with a generalised scarcity of information in the literature regarding marketed products and the limited offer of nutritious and low FODMAP foodstuffs in general^(6)^. This constitutes an obstacle in following a low FODMAP diet^(6,11)^, since the consumer not only consumes natural food but increasingly seeks different industrially accessible options available on the market.

In addition, there has been a significant increase in the demand for gluten- and lactose-free foodstuffs, both in Portugal and in Europe in the last years^(12–15)^. The foodstuffs with the nutritional claim gluten-free and/or lactose-free are not necessarily low in FODMAP content^(16,17)^, as they have not been formulated for this purpose. Gluten is a group of proteins, whereas FODMAP are fermentable carbohydrates. Grains and cereals such as wheat contain both gluten and FODMAP (especially fructans), while most gluten-free grains are naturally low in FODMAP^(16–18)^. This overlap may explain the improvement in symptoms reported by some individuals when excluding gluten from their diet, which could be related to a reduction in FODMAP intake rather than to the removal of gluten itself ^(16,17,19)^. Moreover, excluding both gluten and lactose from the diet can improve general GI symptoms, particularly those with intolerance or sensitivity to these components^(19)^. Therefore, this study aims to estimate the FODMAP content of a sample of industrial pre-packaged food targeted to lactose and/or gluten intolerances.

## Methods

This cross-sectional study focused on products with ‘gluten-free’ and/or ‘lactose-free’ claims. Data were extracted from a database of approximately 8700 food products sold by a market-leading retail chain in Portugal during 2020 and 2021^(20)^. A total of 499 products with the above-mentioned claims were identified. After eliminating duplicated products and verifying that some products did not contain a complete ingredient list (*n* 63), the final sample of this observational study consisted of 436 products.

The ingredient list (in descending order of weight), the content of carbohydrates and sugars per 100 g of product, the net weight and the serving size in grams (if indicated on the packaging) of the selected products were collected. When available, the percentages of the listed ingredients were also recorded to allow for quantitative estimations. The study sample was distributed across twelve food categories: (1) dairy products; (2) plant-based alternatives; (3) biscuits and crackers; (4) desserts and pastries; (5) grocery (e.g. canned foods, grains, fruit pouches, snacks and energy bars); (6) sauces, dressings, creams and soups; (7) ready meals; (8) ice creams; (9) frozen products; (10) sweeteners, gums and chocolates; and (11) bread and toasts and (12) charcuterie. For a uniform analysis, the net weight of all products was converted from millilitres to grams. The ingredient list of eligible products was analysed, identifying possible FODMAP sources described in the literature, namely, fructose, fructans, galactooligosaccharides, lactose and polyols^(21,22)^, and specifically described in the Regulation (EU) No.1169/2011^(23)^ for polyols. When available, the quantity of the described ingredients was estimated by net weight, per 100 g of product and serving size. After the product-by-product enquiry, each product was classified as ‘low FODMAP’, ‘high FODMAP’ or ‘unknown FODMAP’ content. For this classification, we used as reference the FODMAP cutoff values published by Varney, Barrett^(16)^ (< 0·30 g per serving of oligosaccharides (grain products, pulses, nuts and seeds) and < 0·20 g per serving (vegetables, fruits and all other food products) – including fructans and oligosaccharides; < 0·15 g per serving of fructose or < 0·40 g when is only FODMAP present; < 0·40 g per serving of total polyols or < 0·20 g only sorbitol or mannitol; < 1·0 g per serving of lactose)^(2,16)^ and the recommended low FODMAP serving sizes available in the Monash University Low FODMAP diet App^(8,22)^. Thus, we applied the following classification:

- Low in FODMAP: if all the ingredients on the list were low in FODMAP.
- High in FODMAP: for cases when the product contained one or more ingredients potentially high in FODMAP with an amount higher than the cutoff values or the recommended serving sizes.
- Unknown FODMAP content: for cases when the product had one or more ingredients potentially high in FODMAP equal to or higher than the cutoff values or recommended serving sizes in the App, but the exact amount was unknown.

Descriptive statistics and the Pearson *χ* ^2^ test were performed to compare proportions of foods across FODMAP groups and food categories, using IBM SPSS Statistics (Version 29.0).

## Results

**Fig. 1.**
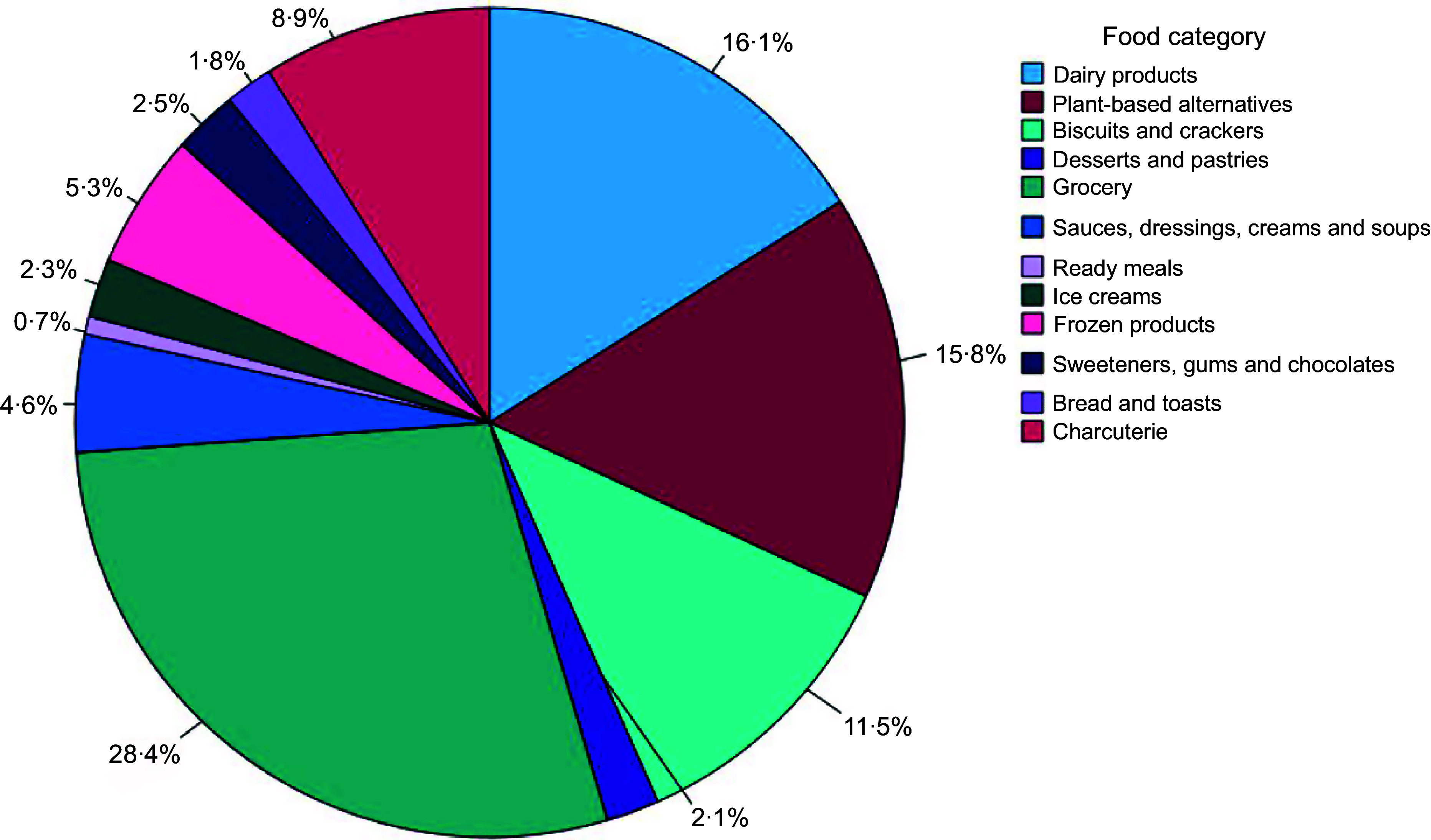
Sample distribution by food category (%) (*n* 436).

The sample consisted of 436 food products (81 white-labelled and 355 from known manufacturers), of which: 61·7 % (*n* 269) contained the nutrition claim ‘gluten-free’; 17·0 % (*n* 74) ‘lactose-free’; and 21·3 % (*n* 93) both nutrition claims. The sample was distributed across twelve food categories, as described in Fig. 1.

The two ingredients most frequently identified in the ingredient lists were ‘soybeans’ (*n* 33; e.g. canned foods, yogurts and plant-based drinks) and ‘garlic’ (*n* 21; e.g. canned foods, snacks, sauces and charcuterie products).

The ingredients with higher amounts per serving than recommended were agave, apple, carob, cashew, celery, chickpeas, chicory root, cocoa, date, fava beans, fructose, fructose syrup, garlic, hazelnuts, honey, inulin, inverted sugar syrup, lactose, lentils, linseed, maltitol (E965), mango, oligofructose, onion, peanuts, pear, peas, pineapple, pumpkin seeds, raisins, sesame, sorbitol (E420), soybeans, sugar, sweet potato, wheat and xylitol (E967). In fact, the ingredient ‘soybeans’ was most frequently observed in ‘plant-based alternatives’ (*n* 25; 75·8%), with an average mass of 9·7 ± 4·3 % as an ingredient and a wide range of sd from 5·2 to 26·0%. A detailed description of the frequencies of all of the ingredients observed in foodstuffs by food categories is provided in the online Supplementary Table S1.

In 29·5 % of products (*n* 128), it was possible to calculate the exact amounts of ingredient sources of FODMAP per net weight, per 100 g of product and for the recommended serving size provided by the manufacturer. In the entire sample, most of the foodstuffs were classified as low (53·0 %, *n* 231); 24·3 % as high (*n* 106) and 22·7 % with unknown FODMAP content (*n* 99).

Statistically significant differences in the proportion of foods based on the FODMAP classification according to food categories were observed (*P* < 0·01). The food category with the highest proportion of products low in FODMAP was ‘dairy products’ (*n* 61; 87·1 %) (Table 1). We can observe that ‘sweeteners, gums and chocolates’ (*n* 4; 36·4 %) and ‘plant-based alternatives’ (*n* 25; 36·2 %) were the two food categories with the highest proportion of products with unknown FODMAP content, which had, however, ingredients that are recognised FODMAP sources. The ‘ready meals’ category (*n* 2; 66·7 %) and ‘sauces, dressings, creams and soups’ (*n* 11; 55·0 %) had the highest proportion of food products with high FODMAP sources.

**Table 1.** Distribution of FODMAP classification by food categories

	Total	FODMAP classification					
		Low FODMAP		High FODMAP		Unknown	
	*n*	*n*	%	*n*	%	*n*	%
Food category							
Biscuits and crackers	50	33	66·0	3	6·0	14	28·0
Bread and toasts	8	6	75·0	1	12·5	1	12·5
Charcuterie	39	20	51·3	7	17·9	12	30·8
Dairy products	70	61	87·1	4	5·7	5	7·1
Desserts and pastries	9	3	33·3	4	44·4	2	22·2
Frozen products	23	11	47·8	8	34·8	4	17·4
Grocery	124	50	40·3	45	36·3	29	23·4
Ice creams	10	4	40·0	4	40·0	2	20·0
Plant-based alternatives	69	30	43·5	14	20·3	25	36·2
Ready meals	3	0	0·0	2	66·7	1	33·3
Sauces, dressings, creams and soups	20	9	45·0	11	55·0	0	0·0
Sweeteners, gums and chocolates	11	4	36·4	3	27·3	4	36·4
Total	436	231		106		99	

FODMAP, fermentable carbohydrates, oligosaccharides, disaccharides, monosaccharides and polyols.

Regarding the frequency of ingredients recognised as sources of FODMAP, fructans were listed in ninety-one products, fructose in fifty-one, galactooligosaccharides in sixty-seven, lactose in twenty-three and polyols in fifty-four. Of these latter fifty-four products, sorbitol was listed in forty-five (83·3 %) products, as a food additive (E420) (*n* 15) or due to its natural presence (*n* 35) (such as apples and pears). Several products contained more than one FODMAP ingredient (e.g. beans (galactooligosaccharides), onion (fructans) and garlic (fructans).

Statistically significant differences were observed for the proportion of foods based on the FODMAP classification according to nutritional claims (*P* < 0·01). We observed that the proportion of ‘high FODMAP’ products in the ‘gluten-free’ and ‘lactose-free’ products was 31·6 and 12·2 %, respectively (Table 2). Globally, it corresponded to 24·0 % of the products included in this study (106 out of 436).

**Table 2.** Distribution of FODMAP classification by nutrition claims

		FODMAP classification							
		Low FODMAP		High FODMAP		Unknown		Total	
		*n*	%	*n*	%	*n*	%	*n*	%
Nutrition claims	Gluten-free	134	49·8	85	31·6	50	18·6	269	
	Lactose-free	61	82·4	9	12·2	4	5·4	74	
	Both	36	38·7	12	12·9	45	48·4	93	
	Total	231	53·0	106	24·3	99	22·7	436	100·0

FODMAP, fermentable carbohydrates, oligosaccharides, disaccharides, monosaccharides and polyols.

## Discussion

More than half of the eligible foodstuffs were classified as low and about a quarter as high in FODMAP, considering the recommended amounts for IBS patients^(16,22)^. This study shows that not all gluten-free and lactose-free products are low in FODMAP and, consequently, they may trigger GI symptoms when consumed. Although people with IBS often choose gluten-free options, it is important to underscore that gluten is not a FODMAP. However, many grain and cereal foods are high in FODMAP (particularly fructans), which can trigger symptoms. Gluten-free and lactose-free products do not guarantee low FODMAP, especially if other high sources of FODMAP (e.g. fructose in bread) have been added^(16,17)^.

Foodstuffs with unknown FODMAP content also represent a sizeable proportion, with ‘sweeteners, gums and chocolates’ and ‘plant-based alternatives’, the categories with the highest proportion of unknown content FODMAP products. The ‘dairy products’ category had a higher proportion of low FODMAP products than the ‘plant-based alternatives’ category. This is not surprising, as lactose-free dairy products have had lactose – a FODMAP – removed. In contrast, plant-based alternatives often list ‘soybeans’ among their ingredients. Soybeans are naturally high in FODMAP, particularly galactooligosaccharides, which may trigger GI symptoms in patients with IBS^(24)^. However, further research is needed to determine the portions that can be tolerated per meal and the appropriate percentages to be listed as ingredients in foodstuffs, as we observed a wide range of ‘soybeans’ percentages in ingredient lists. Furthermore, more information on the production process of industrial foods is needed since pulses and sprouted grains may have different FODMAP content, depending on the production process^(6,25)^.

In the ‘grocery’ category, a wide variety of foodstuffs was observed, In the ‘grocery’ category, a wide variety of foodstuffs was observed, namely, breakfast cereals (*n* 14), canned foods (*n* 5), energy bars (*n* 28), fruit pouches (*n* 17), grains (*n* 43) and snacks (*n* 17). Some products were classified as low in FODMAP according to the recommended serving size indicated by the supplier. However, it may be important to check with patients the actual amount usually consumed, as some breakfast cereals may be ingested in larger quantities than those suggested on the packaging. Even the energy bars had different net weights (25 g, 40 g, 120 g and 160 g), which may influence the portions consumed per meal. Possibly, the lower portions (25 g and 40 g) may be tolerable and thus recommended for the first phase of the low FODMAP diet. We believe that some products in the ‘grocery’ category could be consumed during the first phase of the low FODMAP diet, since FODMAP content may vary depending on serving size, food preparation and even the ripeness of certain foods, such as fruits^(26)^. Nevertheless, it is necessary to determine both the serving size and the exact FODMAP content.

The serving size per meal is crucial in manifesting GI symptoms, reinforcing the importance of better transparency regarding the recommended serving sizes of foodstuffs. We also found that, within the fruit pouches for both children and adults, apple accounted for an average of more than 50 % of the product composition, even when mixed with other low FODMAP fruits (57·3 ± 19·4 %; minimum 30·0 % and maximum 79·9 %). This may be of concern for Portuguese IBS patients, as apples are among the fruits with the highest availability for consumption in Portugal (24·9 kg/capita per year)^(27)^.

There were a few ‘ready meals’ containing gluten or lactose-free claims, but none was low in FODMAP. These data show the scarcity of the supply for these types of products in the Portuguese market adapted for IBS patients, being one of the most relevant difficulties reported by these patients, including the limited food options when eating out^(16)^. A similar scenario was observed for ‘desserts and pastries’, ‘sweeteners, gums and chocolates’, ‘bread and toasts’ and ‘ice creams’, which showed to have a limited offer of low FODMAP options.

Sorbitol (E420) was identified in the ingredient list of several charcuterie products, being used as a stabiliser. Other stabilisers were also observed in this category, such as carrageenan (E407) and guar gum (E412), whose contribution to the risk of developing GI symptoms is lower in IBS patients^(23)^. The E-numbers that were observed in the ingredient lists of the study sample may help formulate new products or reformulate existing ones to reduce FODMAP content. Sorbitol was also the most observed polyol, both used as a food additive (E420) or present in foods naturally containing this compound (e.g. apples). These data support the information that sorbitol is the most consumed polyol and the most used non-nutritive sweetener^(28–30)^.

Alongside the selection of ingredients in processed and ultra-processed products, it is also important to verify the processing techniques used, as these can alter the FODMAP content^(6,16)^. Additionally, it is important to consider possible international and regional differences in the content of FODMAP due to the agricultural production, as well as the different techniques used in product processing and ingredient selection^(16)^. Many patients incorrectly self-diagnose themselves as gluten and lactose intolerant because they believe that these nutrients are associated with GI symptoms, leading them to avoid their consumption^(19)^. Information is scarce in the literature regarding marketed products and the limited availability of nutritious, low FODMAP options. Our study aimed to highlight possible high FODMAP ingredients in foodstuffs through estimation. However, only direct analytical testing could verify whether the product meets published cutoff values.

Although food labels in Europe indicate the total content of carbohydrates, sugars and fibres, FODMAP are not indicated on the labels, not allowing them to be easily identified^(11,21,26)^. However, it is possible to identify polyols in the list of ingredients. Thus, it is necessary to inform the patient about the E-numbers used since in most of the lists observed in this study, polyols stood out only for their E-numbers. A possible suggestion for the future would be the mandatory inclusion of the polyol content in the nutritional declaration of foodstuffs commercialised in the European Union (EU), provided by the Regulation (EU) No.1169/2011^(23)^, in case the product has these ingredients. These data would not be indicators of the total FODMAP components present in a product, but their inclusion will facilitate the choice of products by IBS consumers, especially those with intolerance to polyols.

Another strategy could be the presence of a logo or a ‘low in FODMAP’ claim, indicating the products are low in FODMAP, which has already been in practice in other countries, like Australia, through food certification programmes for this purpose. To date, and to our best knowledge, there are two certification programmes for low FODMAP products, both located in Australia, namely, the ‘Monash University Low FODMAP Certification Program’ (www.monashfodmap.com) and ‘The FODMAP Friendly Certification Program’ (www.fodmapfriendly.com), which analytically assess the FODMAP content in products, listing them on their apps and websites so that the consumer can be up to date^(6,11)^.

While the concept of FODMAP is well defined and regulated by the Australian Food Standards, with products approved under certification programmes, this is not the case in the EU^(6,25)^. A recent study reported that only 19 % of low FODMAP products were available in the EU, and the majority had no information about being a low FODMAP product, which highlights the lack of definition and non-existent regulation^(6,25)^. The creation of a unique logo or claim could be a solution to allow an easy identification of these products globally, based on a standardised scientific methodology for analysing the content of FODMAP in products^(11)^ could significantly impact life quality of IBS patients. We believe that the criteria applied in Australian certification programmes may be incorporated into future European regulations, similar to other existing ones such as the Regulation (EU) No.1169/2011^(23)^, which provides for the concepts of nutrition and health claims, or even the Regulation (EU) No 828/2014^(31)^, which establishes requirements for the provision of information on food allowing consumers to identify products with absence or reduced content of gluten^(6)^.

Both manufacturers, academic institutions, regulators and health professionals working on nutrition and gastroenterology should seek to work together to present more appropriate and easily identifiable low FODMAP solutions for patients^(6,11)^.

## Conclusion

Only 53 % of the products with ‘gluten-free’ and/or ‘lactose-free’ claims were low in FODMAP. This study highlights the importance of clearly identifying the sources of FODMAP used in foodstuffs targeted to GI pathologies in Portugal. Laboratory studies should be performed to better characterise the FODMAP content in processed products chemically, to establish adequate and precise serving sizes per meal, to improve the information regarding the presence of ingredients high in FODMAP as well and to define the formulation of new foodstuffs with low FODMAP.

### Strengths and limitations

First, the sample of products used in this study was obtained from a single food retail chain, which may limit the representativeness of our results. However, this chain is a market leader in Portugal (market share > 20 %), has national coverage and offers products from a wide range of manufacturers, many of which are also available in other national supermarkets, thereby increasing the generalisability of our findings. Second, our study estimated the possible FODMAP content in foodstuffs based on their ingredient lists, which did not allow the quantification of all ingredients potentially high in FODMAP. Nevertheless, to the best of our knowledge, this is the first Portuguese study to comprehensively identify FODMAP sources in the ingredient lists of foods targeted to GI pathology. It highlights key ingredients present in foodstuffs commercialised in Portugal, underscores the need for nutrition education and awareness among dietitians and points to the importance of improving labelling information to empower IBS consumers in Europe.

## Supporting information

Barreirinhas et al. supplementary material Barreirinhas et al. supplementary material

## Supplementary material

For supplementary material/s referred to in this article, please visit https://doi.org/10.1017/S000711452510528X.
