# Supplementary material for: Pre-packaged food targeted to gastrointestinal pathologies: are they low in FODMAP?
Source: Br J Nutr. 2025 Oct 9;134(9):727–32. doi: 10.1017/S000711452510528X (PMC12766106; doi:10.1017/S000711452510528X)
Supplement: Barreirinhas et al. supplementary material [file S000711452510528Xsup001.docx]

| Table S1. Ingredients observed in foodstuffs by food categories | | | | | | | | | | | | | |
| --- | --- | --- | --- | --- | --- | --- | --- | --- | --- | --- | --- | --- | --- |
| Ingredients | *Dairy products*  *n (%)^1^* | *Plant-based alternatives*  *n (%)^1^* | *Biscuits and crackers*  *n (%)^1^* | *Desserts and pastries*  *n (%)^1^* | *Grocery*  *n (%)^1^* | *Sauces, dressings, creams and soups*  *n (%)^1^* | *Ready meals*  *n (%)^1^* | *Ice creams*  *n (%)^1^* | *Frozen products*  *n (%)^1^* | *Sweeteners, gums and chocolates*  *n (%)^1^* | *Bread and toasts*  *n (%)^1^* | *Charcuterie*  *n (%)^1^* | Total  *n^2^* |
| *Agave* | - | - | - | - | 4 (100.0) | - | - | - | - | - | - | - | **4** |
| *Apple* | - | - | - | - | 18 (100.0) | - | - | - | - | - | - | - | **18** |
| *Broad beans (favas)* | - | 2 (100.0) | - | - | - | - | - | - | - | - | - | - | **2** |
| *Carob* | 1 (12.5) | 2 (25.0) | - | 1 (12.5) | - | - | - | - | - | - | 1 (12.5) | 3 (37.5) | **8** |
| *Cashew* | - | - | - | - | - | 1 (100.0) | - | - | - | - | - |  | **1** |
| *Celery* | - | - | - | - | - | - | - | - | - | - | - | 3 (100.0) | **3** |
| *Chickpeas* | - | - | - | - | 3 (60.0) | - | - | - | 2 (40.0) | - | - | - | **5** |
| *Chicory root* | - | 4 (36.4) | - | - | 4 (36.4) | - | - | 3 (27.3) | - | - | - | - | **11** |
| *Cocoa* | - | - | 1 (16.7) | - | 4 (66.7) | - | - | - | - | 1 (16.7) | - | - | **6** |
| *Date* | - | - | - | - | 16 (100.0) | - | - | - | - | - | - | - | **16** |
| *Fructose* | - | 3 (42.9) | 1 (14.3) | - | 2 (28.6) | - | - | 1 (14.3) | - | - | - | - | **7** |
| *Garlic* | - | - | - | - | 3 (14.3) | 7 (33.3) | 1 (4.8) | - | 5 (23.8) | - | - | 5 (23.8) | **21** |
| *Fructose syrup* | - | - | - | - | 1 (33.3) | - | - | 1 (33.3) | - | 1 (33.3) | - | - | **3** |
| *Hazelnuts* | - | - | - | - | - | - | - | - | - | 1 (100.0) | - | - | **1** |
| *Honey* | - | - | 2 (33.3) | - | 4 (66.7) | - | - | - | - | - | - | - | **6** |
| *Inulin* | 4 (28.6) | 5 (35.7) | 1 (7.1) |  | 2 (14.3) |  |  |  |  | 2 (14.3) |  | - | **14** |
| *Inverted sugar syrup* | - | - | 3 (75.0) | - | - | - | - | - | - | 1 (25.0) | - | - | **4** |
| *Lactose* | - | - | 2 (11.8) | 1 (5.9) | 6 (35.3) | - | 1 (5.9) | 3 (17.6) | 2 (11.8) | - | - | 2 (11.8) | **17** |
| *Lentils* | - | - | - | - | 3 (100.0) | - | - | - | - | - | - | - | **3** |
| *Linseed* | - | - | - | - | 2 (66.7) | - | - | - | - | - | 1 (33.3) | - | **3** |
| *Maltitol* | - | - | 1 (20,0) | - | 1 (20.0) | - | - | 1 (20.0) | - | 2 (40.0) | - | - | **5** |
| *Mango* | - | - | - | - | 1 (100.0) | - | - | - | - | - | - | - | **1** |
| *Oligofructose* | 5 (50.0) | - | 3 (30.0) | - | 2 (20.0) | - | - | - | - | - | - | - | **10** |
| *Onion* | - | - | - | - | 2 (11.1) | 9 (50.0) | 1 (5.6) | - | 2 (11.1) | - | - | 4 (22.2) | **18** |
| *Peanuts* | - | - | - | - | 3 (100.0) | - | - | - | - | - | - |  | **3** |
| *Pear* | - | - | - | - | 8 (100.0) | - | - | - | - | - | - | - | **8** |
| *Peas* | - | 4 (30.8) | - | - | 8 (61.5) | - | - | - | 1 (7.7) | - | - | - | **13** |
| *Pineapple* | - | - | - | - | 1 (100.0) | - | - | - | - | - | - | - | **1** |
| *Pumpkin seeds* | - | - | - | - | 1 (100.0) | - | - | - | - | - | - | - | **1** |
| *Raisins* | - | - | - | - | 1 (100.0) | - | - | - | - | - | - | - | **1** |
| *Sesame* | - | - | - | - | 5 (100.0) | - | - | - | - | - | - | - | **5** |
| *Sorbitol (E420)* | - | - | - | - | - | - | - | - | 4 (26.7) | 1 (6.7) | 1 (6.7) | 9 (60.0) | **15** |
| *Soybeans* | - | 25 (75.8) | - | 2 (6.1) | 2 (6.1) | - | - | - | - | - | - | 4 (12.1) | **33** |
| *Sugar* | - | - | - | 3 (33.3) | 1 (11.1) | - | - | - | - | 5 (55.6) | - | - | **9** |
| *Sweet potato* | - | - | - | - | 1 (100.0) | - | - | - | - | - | - | - | **1** |
| *Wheat* | - | 2 (50.0) | 1 (25.0) | - | - | - | - | - | 1 (25.0) | - | - | - | **4** |
| *Xylitol (E967)* | - | - | - | - | 2 (100.0) | - | - | - | - | - | - | - | **2** |
| Total | **10** | **47** | **15** | **7** | **111** | **17** | **3** | **9** | **17** | **14** | **3** | **30** |  |

^1^ n = number of times each ingredient was observed by category in absolute value; (%) = number of times each ingredient was observed in percentage; ^2^ n = total number of times each ingredient was observed
